# Reduced ovarian reserve in young early breast cancer patients: preliminary data from a prospective cohort trial

**DOI:** 10.1186/s12885-017-3593-x

**Published:** 2017-09-06

**Authors:** Antonia Wenners, Jana Grambach, Juliane Koss, Nicolai Maass, Walter Jonat, Andreas Schmutzler, Christoph Mundhenke

**Affiliations:** 1 OB/GYN, University of Kiel, UKSH, Arnold-Heller-Straße 3, 24105 Kiel, Germany; 2 OB/GYN, Reproductive Medicine, University of Kiel, UKSH, Arnold-Heller-Straße 3, 24105 Kiel, Germany

**Keywords:** Fertility preservation, Ovarian reserve, Breast cancer, AMH

## Abstract

**Background:**

The numerous side effects of chemotherapy in patients with breast cancer are well known. However, the precise effects of chemotherapy on ovarian function in premenopausal women are poorly investigated. The patients are at risk of developing sexual hormone deficiency and impaired fertility. This prospective cohort study addresses predictive parameters of ovarian reserve after chemotherapy.

**Methods:**

Fifty-one premenopausal women (28–46 years) with primary breast cancer were included in the trial. All of them received anthracycline-based chemotherapy (*n* = 18), or combinations with taxanes (*n* = 30), or anthracycline-free chemotherapy (*n* = 3). Changes in hormone levels (LH, FSH, E2 and Anti-Müllerian hormone (AMH)), antral follicle count (AFC), and amenorrhea were determined before (V1), and 6, 12 and 24 months after the initiation of chemotherapy (V2-V4). Quality of life parameters were evaluated. The additional impact of parity, BMI, and smoking on ovarian reserve was also assessed.

**Results:**

AFC and AMH fell very markedly after chemotherapy and did not return to pre-treatment levels until V4. A significant positive correlation was noted in AFC before and 1 year after chemotherapy. AMH levels at V2-V4 were significantly correlated with those registered at V1. AFC and AMH were negatively correlated with age. Continued smoking had a significant detrimental effect on AFC after 24 months. LH and FSH levels increased between V1 and V2 and fell at V3 and V4, but stayed above pre-chemotherapy values. Two years after the start of chemotherapy 31/51 patients were amenorrhoic while 17 resumed their menstrual cycle; this was not influenced by the type of chemotherapy or age. Non-smokers were 13 times more likely to resume their menstruation than smokers. Quality of life (QL) was significantly lower 6 months after the initiation of chemotherapy. QL at one and 2 years after chemotherapy did not differ significantly from pre-chemotherapy scores.

**Conclusions:**

Our study contributes to a better understanding and prediction of ovarian reserve in young early breast cancer patients undergoing chemotherapy. The data suggest that personal counseling in regard of the preservation of fertility should be offered especially to patients of a higher age, with low AMH levels or low follicle counts. Patients should be advised to stop smoking in order to enhance the likelihood of preserving their fertility.

**Electronic supplementary material:**

The online version of this article (10.1186/s12885-017-3593-x) contains supplementary material, which is available to authorized users.

## Background

Eight percent to 12% of all breast cancers occur before the age of 35 years [1]. The side effects of chemotherapy are well known. Nevertheless, we lack knowledge of their precise effects on ovarian function and fertility. Adjuvant chemotherapy is indicated for patients with a high risk of recurrent disease [2]. Endocrine therapy is recommended for all tumors that express the estrogen (ER) and/or progesterone receptor (PR). Tamoxifen is a selective estrogen receptor modulator and is the endocrine therapy of choice in premenopausal women (20 mg per day for 5 to 10 years) [3]. In premenopausal patients with ER/PR-positive high-risk cancers or patients aged <35 years, gonadotropin-releasing hormone (GnRH) analogs/agonists may be used in addition to tamoxifen [4–6].

In young patients the treatment of breast cancer is rendered difficult by the fact that they may still wish to have children [7]. Therefore, issues such as fertility preservation and planning a pregnancy after the conclusion of chemotherapy should be addressed prior to the start of treatment. However, many patients are much older when their treatment is concluded, and ovarian function is additionally harmed by chemotherapy and endocrine therapy [1]. Symptoms of sexual hormone deficiency, chemotherapy-induced amenorrhea (CIA), and impaired fertility are known to occur [8]. CIA begins shortly after the first dose of chemotherapy, persists up to several years after the conclusion of chemotherapy, and possibly continues directly into menopause.

Ovarian function is measured by hormone levels in the early phase of the menstrual cycle. This includes estrogen (E2), follicle-stimulating hormone (FSH), luteinizing hormone (LH), prolactin, testosterone and Anti-Müllerian hormone (AMH) on day 3–5 of the cycle [9]. Postmenopausal hormone levels are reached when the ovaries are resistant to gonadotropins and when LH levels are 5-fold above normal. FSH is elevated 10- to 15-fold andestradiol decreases below 20 pg/ml. [9]. AMH plays a special role because it remains nearly unaltered by exogenous steroids or hormonal contraception and cyclic fluctuations [10]. A transvaginal ultrasound may be performed to determine the number of antral follicles, endometrial thickness, and measure the volume of the ovaries [1]. AMH is fundamentally involved in folliculogenesis and the selection of a dominant follicle by inhibiting FSH. Currently it is one of the most important parameters to measure ovarian reserve [11, 12] and is strongly correlated with the antral follicle count [13, 14]. AMH levels and age, as well as AMH levels and body mass index are negatively correlated [10, 15]. AMH values may vary significantly, depending on individual biological fluctuations, exposure to medication, ovarian surgery, and different detection assays [16]. The standard reference value for AMH at our institution is 2.0–6.8 ng/ml (Table 1). Estradiol (range 10–500 pg/ml) is formed in the granulosa cells of a maturing follicle and after ovulation in the corpus luteum [9]. Women produce small quantities of the androgen testosterone in stromal cells of the ovary and the adrenal cortex [9].

**Table 1:** Local reference values

	25–29 years	30–34 years	35–39 years	40–44 years	40–50 years
AMH ng/ml	1.18–9.16	0.67–7.55	0.78–5.24	0.10–2.96	0.05–2.06
	Luteal	Ovulation	Follicular	Menopause	
Estradiol ng/ml	43.8–211	85.8–498	12.5–166	< 54.8	
FSH IU/l	1.7–7.7	4.7–21.5	3.5–12.5	25.8–134.8	
LH IU/l	1–11.4	14–95.6	2.4–12.6	7.7–58.5	

Sixty-nine percent of all changes in the volume of the ovaries are age related. The volume fluctuates between 0.7 ml at the age of 2 years, to 7.7 ml at the age of 20 years and 2.8 ml at the time of menopause. In addition to AMH and AFC, the volume of the ovary is a useful biomarker of ovarian reserve [17]. Chemotherapy and tamoxifen may reduce the volume [18]. The number of primordial follicles decreases in the course of a woman’s life: 400,000 follicles are present at puberty and a mere 70,000 at the age of 40 years [1]. Chemotherapy significantly enhances the degradation of primordial follicles in premenopausal women [19]. The gonadotoxicity of chemotherapy drugs is very diverse [20].

Pharmacological protection of the ovaries by GnRH analogs and active reproductive methods such as cryopreservation of fertilized or unfertilized oocytes have been discussed in the past. [21]. An innovative and experimental alternative is ovarian tissue freezing and transplantation [22].

The changes in a young woman’s life during and after chemotherapy require adequate counseling in addition to oncologic care. In the present study we evaluate the precise effects of different chemotherapy drugs on the ovarian function and fertility of young breast cancer patients. When a patient’s data indicate a high risk of infertility, it would be meaningful to consider methods for the preservation of ovarian function or fertility, such as cryopreservation of oocytes or embryos, prior to the initiation of chemotherapy. Predictive parameters of ovarian reserve after chemotherapy could be helpful in providing supportive cancer therapy for premenopausal breast cancer patients.

## Methods

A prospective cohort study was performed at a single academic breast cancer center. Data were collected from March 2010 to May 2014, with a follow-up period of 24 months. Fifty-one premenopausal women were enrolled in the study before the initiation of (neo-)adjuvant chemotherapy for primary breast cancer. All patients had given their written informed consent and the study was approved by the local review board (AZ D 432/09, 21.09.2009).

At each visit the patients underwent a transvaginal ultrasound investigation, blood samples were taken, and three questionnaires were answered by the patients. The first visit (V1) took place before the first administration of chemotherapy. The second visit (V2) occurred 6 months, the third (V3) 12 months, and the fourth (V4) 24 months (22–26 months) after the initiation of chemotherapy. Blood samples were analyzed in regard of AMH, E2, FSH, LH, prolactin and testosterone at each visit, with an electrochemiluminescence immunoassay (ECLIA) using Cobas8000 Module602 (Roche Diagnostics).

Ultrasound was performed by experienced physicians. In addition to endometrial thickness and ovarian volume (on either side), the number of antral follicles in the ovaries were determined. The ovaries were screened in two planes for follicles (2–10 mm), which were counted separately for each side [14]. Intra-cycle variability is minimized by performing the AFC on the 3rd to 5th day of the cycle. This optimal timing was not always possible (at visit 1) because chemotherapy had to be started immediately in some patients. It was also impossible to find an optimal day of the cycle in amenorrhoic women. Chemotherapy-induced amenorrhea (CIA) was defined as amenorrhea within 6 months after the initiation of chemotherapy. Self-reported menstrual histories were used to determine clinical ovarian function regardless of hormone levels. Resumption of the menstrual cycle was defined as repeated cyclic bleeding after CIA and conclusion of chemotherapy, during the follow-up period of 24 months.

Quality of life (QL) was evaluated by three questionnaires which were filled by the patients at each of the four visits: the EORTC QLQ-C30 (core module) [23], the EORTC QLQ-BR23 (breast cancer module) [23], and a special questionnaire designed for the study (see copy as Additional file 1). Standardized algorithms were used for the evaluation of QL [24]. All of the scales and single-item measures ranged from 0 to 100. A high score signified a higher response level. We also used a special questionnaire generated for the study, with questions on age, marital status, parity and children, education, pre-existing medical conditions, medication, smoking and alcohol consumption, the ongoing desire to have children, history of miscarriage and abortions, contraceptive methods used, the duration of the menstrual cycle, age at menarche, and when amenorrhea occurred during or after chemotherapy. QL data were then correlated with data from transvaginal ultrasound and laboratory results. Data concerning the current cancer were recorded, including adjuvant or neoadjuvant therapy, chemotherapy regimen and dose, endocrine therapy, and body mass index (BMI).

### Statistical methods

Quantitative values were presented as means and standard deviations, minimum and maximum, as well as quartiles. The data were tested for normal distribution using the Kolmogorov-Smirnov test in case of significant deviations from normal distribution. The Kruskal-Wallis test was used to compare more than two independent samples, and the Mann-Whitney U test for two independent samples. Two measurements over time were compared with Wilcoxon matched pairs test each, and correlation analysis was performed using Spearman’s rank correlation. Parametric methods were used in the absence of significant deviations from normal distribution. Two independent samples were compared with the two-sample t-test.

Ordinal and nominal values were shown in absolute and percent frequencies. Two of each of these values were compared in contingency tables and tested for dependence with the chi-square test. Fisher’s exact test was used when the expected frequencies proved to be too small. Odds ratios were calculated to quantify the effect of the influencing factors.

The tests were two sided with a significance level of 5%. An alpha adjustment for multiple testing was not performed, and the results were interpreted accordingly. SPSS Statistics 22 (SPSS Inc. an IBM Company, Chicago, IL) was used for statistical calculations.

## Results

The mean age of the 51 women was 38 years (range, 28–46 years) at V1. They received either anthracycline-based “A” chemotherapy (5-fluorouracil, epirubicin, cyclophosphamide (FEC); *n* = 18) or a combination with taxanes “T” (docetaxel, doxorubicin, cyclophosphamide (TAC) or FEC/docetaxel (Doc) or epirubicin, cyclophosphamide (EC)/paclitaxel (Pac); *n* = 30), or an anthracycline-free “AF” regimen (cyclophosphamide, methotrexate, 5-fluorouracil (CMF) or Doc; *n* = 3).Thirty-two patients were ER/PR-positive and received tamoxifen, while 19 were ER/PR-negative. Seven patients received GnRH agonists. Forty-four patients attended all four visits. Four patients died during the study period, one of them after her fourth visit. Four patients withdrew their consent. Patient characteristics are summarized in Table 2.

**Table 2:** Patient characteristics

		Number	Percent
Parity	Nulliparous	18	35.3
	Primiparous	19	37.3
	Multiparous	14	27.4
Smoking	yes	14	27.5
	no	37	72.5
Resumption of the menstrual cycle	yes	19	38.0
	no	31	62.0
Chemotherapy regimen	A	18	35.3
	T	30	58.5
	AF	3	5.9
GnRH analog	yes	7	13.7
	no	44	86.3
Tamoxifen	yes	32	62.7
	no	19	37.3

*A* anthracycline-based, *AT* combination with taxanes, *AF* anthracycline-free

### Markers of ovarian reserve: AFC and AMH

**Fig. 1:**
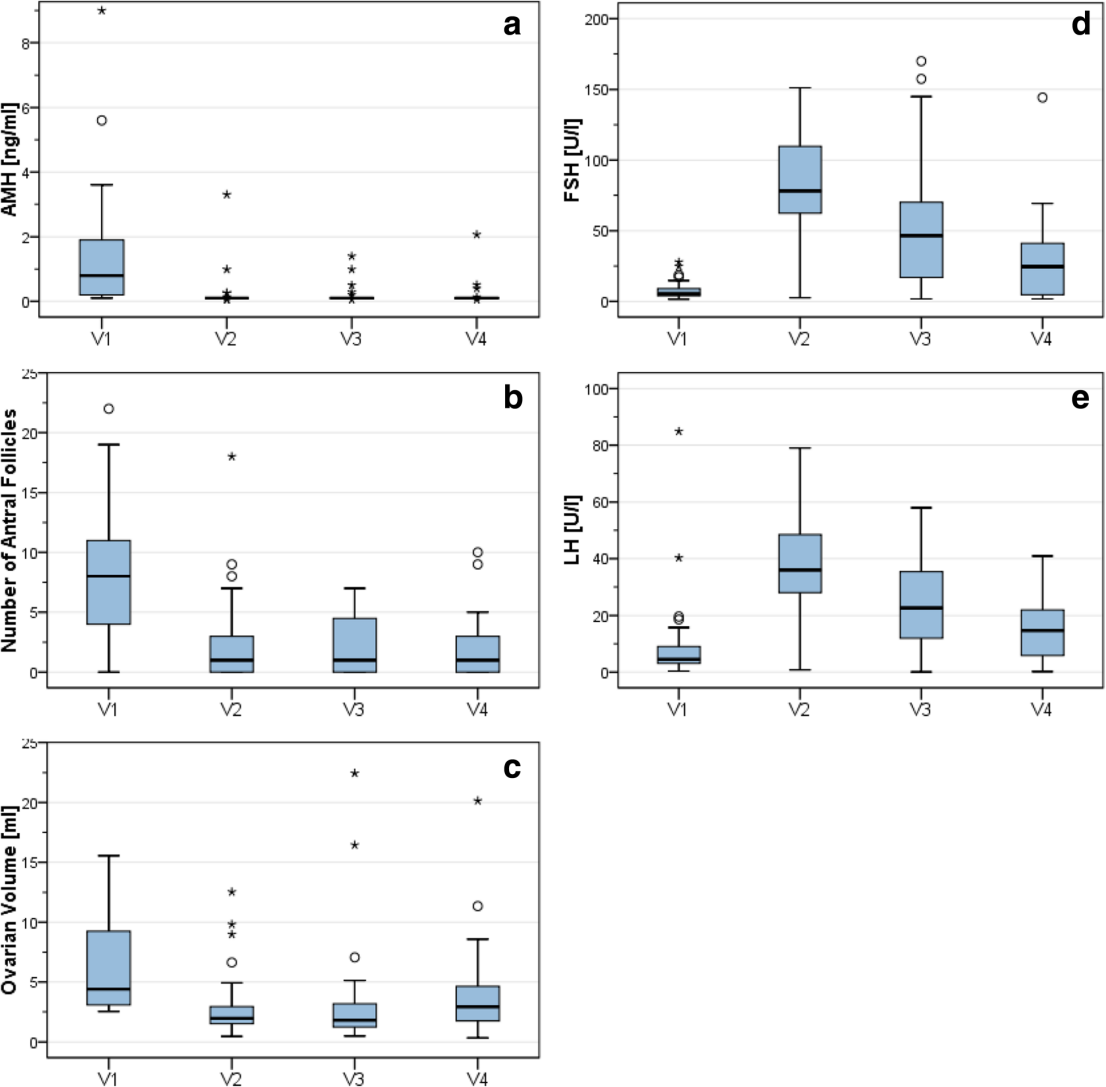
Development over the period of observation. **a** AMH; **b** Number of antral follicles; **c** Ovarian volume; **d** LH; **e** FSH. In the box plots 50% of the values are located within the box. The upward or downward antennas each represent 25% of the values. The horizontal line inside a box indicates the median value. A circle (°) marks outlier values whose spacing down to the 25% or up to the 75% percentile is between 1.5 to 3 times the height of the box. An asterisk (*) indicates extreme values whose distance from the 25% or 75% percentile is more than three times the height of the box [41]

AFC fell very markedly from 8.5 at V1 to 2.1–1.8 at V2–4 after chemotherapy; pre-treatment levels were not achieved until the end of the observation period (Fig. 1b). AFC levels before chemotherapy were significantly positively correlated with those registered 1 year after chemotherapy (*p* = 0.012). AMH levels also fell markedly from V1 to V2–4. AMH levels at V2–4 (0.16–0.19 ng/ml) were correlated with those at V1 (1.28 ng/ml) (V2 *p* = 0.013; V3 *p* = 0.004, V4 *p* = 0.012; Fig. 1a).

Both AFC and AMH were negatively correlated with age at V1 (*p* = 0.004; *p* < 0.001), and AMH at subsequent visits as well (*p* = 0.032 at V4). Neither at V4 nor at other visits did the type of chemotherapy (A vs. T vs. AF) or its cumulative dose influence AFC or AMH levels (both *p* > 0.05). Compared to the absence of endocrine therapy, the use of tamoxifen at V4 was correlated with a significant reduction in AFC (*p* = 0.039), but had no impact on AMH levels. The number of smokers at all 4 visits was constant (*n* = 14). Continued smoking had a significant detrimental effect on AFC after 24 months (*p* = 0.001). BMI showed no significant correlation with AFC or AMH. The data are shown in Table 3.

**Table 3:** Associations with markers of ovarian reserve

	AFC [*p*-value]				AMH [*p*-value]			
	V1	V2	V3	V4	V1	V2	V3	V4
Age [years]	*0.004*	0.326	0.683	0.268	*<0.001*	0.821	*0.008*	*0.032*
Chemotherapy regimen (A vs. T vs. AF)	0.880	0.392	0.120	0.198	0.173	0.428	0.249	0.277
Tamoxifen (yes vs. no)	0.114	0.930	0.953	*0.039*	*0.036*	0.683	0.259	0.119
Smoking (yes vs. no)	0.124	0.292	0.124	*0.001*	0.328	0.789	0.297	0.389
BMI	0.284	0.647	0.713	0.974	0.077	0.487	0.574	0.669

*AFC* antral follicle count, *AMH* Anti-Müllerian hormone, italic numbers present significant *p*-values

### Ovarian size

Ovarian size decreased significantly between V1 (6.4 m^3^) and V2 (2.7 m^3^; *p* = 0.008), but increased thereafter (V3 3.1 m^3^, V4 3.7 m^3^; Fig. 1c).

Ovarian volume was positively correlated with AFC at V4 (*p* = 0.003), but not with AMH. A negative correlation was noted between ovarian size at V4 and LH, FSH and E2 at V1 (*p* = 0.002, *p* = 0.025, *p* = 0.037). Positive correlations with E2 levels were observed at each corresponding visit (*p* < 0.001). Patients who resumed menstruation had significantly larger ovaries at V4 (5.0 m^3^) compared to those who remained amenorrhoic (2.9 m^3^; *p* = 0.007). Smokers had significantly smaller ovaries at V4 (2.8 m^3^) than non-smokers (4.1 m^3^; *p* = 0.027).

No significant association was observed with age, the type or dose rate of chemotherapy, the duration of amenorrhea, and BMI.

### Endocrine markers: LH, FSH, E2

Patients experienced an increase of LH and FSH between V1 (LH 8.9 IU/l, FSH 7.8 IU/I) and V2 (LH 36.5 IU/l, FSH 19.3 IU/I). FSH rose to the postmenopausal level of 47.0 IU/I at V3, whereas LH had started to fall (22.1 IU/l). Both LH and FSH fell further at V4 (LH 14.7 IU/l, FSH 26.9 IU/I), but stayed above pre-chemotherapy levels (Fig. 1d and e). FSH at V4 was correlated with V1 levels (*p* = 0.005), but LH showed no correlation. At V4, LH and FSH were positively correlated with age (*p* = 0.001). E2 showed a negative but non-significant correlation with age. Prolactin and testosterone revealed no correlations with the given data.

LH, FSH and E2 were not influenced by the type of chemotherapy, its cumulative dose, or subsequently by tamoxifen alone compared to patients without endocrine therapy. However, with regard to patients receiving endocrine therapy, a significant difference was noted between those who were treated with tamoxifen alone and those who received both tamoxifen and a GnRH agonist. The first group had significantly higher levels of LH and FSH at V3 and V4 (LH/FSH *p* = 0.002; *p* < 0.001). The combination of tamoxifen and a GnRH agonist differed significantly from both, patients receiving no tamoxifen (LH/FSH *p* = 0.001, *p* = 0.003) and patients receiving tamoxifen alone (LH/FSH *p* ≤ 0.001).

E2 levels at V4 were significantly reduced (*p* = 0.003) by continued smoking, whereas LH and FSH levels were not influenced by smoking.

None of the endocrine markers was significantly correlated with BMI.

### Amenorrhea and resumption of menstruation

All but 3 patients turned amenorrhoic within 6 months after the first administration of chemotherapy. Two years after the initiation of chemotherapy, 31 of 48 patients with CIA remained amenorrhoic while 17 (35.4%) resumed their menstruation. The mean duration of amenorrhea in CIA patients was 20 months (range, 3–26 months).

**Fig. 2:**
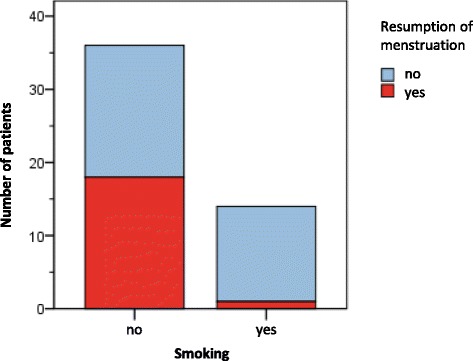
Number of patients who resumed menstruation and their smoking behavior

A significant negative correlation was noted between the duration of amenorrhea and AFC at V1 (*p* = 0.009), V2 (*p* = 0.04) and V3 (*p* = 0.003). Low AMH levels were also correlated with longer amenorrhea at V1 (*p* = 0.014) and V4 (*p* = 0.035). Non-smokers were 13 times more likely to resume their menstruation than smokers. Non-smokers started bleeding again in 50% of cases, whereas 92% of smokers remained amenorrhoic (*p* = 0.005; Fig. 2; Table 4).

**Table 4:** Associations with the duration of amenorrhea and resumption of menstruation

	Duration of amenorrhea [*p*-value]	Resumption of menstruation [*p*-value]
Age [years]	0.332	0.056
Chemotherapy regimen (A vs. T vs. AF)	0.203	0.187
Hormone therapy (yes vs. no)	0.066	0.058
Smoking (yes vs. no)	0.082	*0.005*
BMI	0.698	0.164

Italic numbers present significant *p*-values

The duration of amenorrhea was not influenced by age, the type of chemotherapy, dose rate, tamoxifen, smoking or BMI. Tamoxifen therapy was correlated with a greater likelihood of permanent amenorrhea (*p* = 0.058, n.s.; Table 4).

### Quality of life

Quality of life was significantly lower at 6 months after the initiation of chemotherapy than at V1. At 1 and 2 years after chemotherapy, QL did not differ markedly from pre-chemotherapy levels, but was significantly higher than the score registered immediately after chemotherapy (*p* = 0.001). This was seen not only on the global health status, but also on functional scales. The patients’ role functioning was poorer at V2 than at V1, but improved at 1 and 2 years after chemotherapy (*p* = 0.028). The patients were less emotional (*p* = 0.005) and had poorer social functioning (*p* = 0.01) at V2 than at V1; both of these functions were improved at V3 and V4. Symptoms such as fatigue (*p* = 0.023) and dyspnea (*p* = 0.003) showed the same pattern. Many patients had significantly more financial problems after the end of chemotherapy, but were in better financial condition at 1 and 2 years after chemotherapy (*p* = 0.001).

No significant correlation was noted in regard of the development of physical functioning, cognitive functioning, nausea and vomiting, pain, loss of appetite, constipation, diarrhea, and insomnia at the different visits.

As regards breast-cancer-specific items, the patients’ body image was not as good at V2 as it had been before chemotherapy, but rose again at 1 and 2 years after chemotherapy (*p* = 0.023). Sexual functioning reached its lowest point at 6 months after the start of treatment, but almost returned to pre-treatment levels 6 months later (*p* = 0.003). Sexual enjoyment did not differ significantly. Future perspectives were better 1 year after chemotherapy than before treatment, but reduced again at V4 (*p* < 0.001). Symptoms in the arms were worst at the last visit 2 years after the start of chemotherapy (*p* = 0.018). No significant difference in quality of life was noted in regard of hair loss or breast symptoms.

No correlation was registered between quality of life and markers of ovarian reserve (AFC, AMH), endocrine markers (LH, FSH, E2), amenorrhea, or the resumption of bleeding. Thirty-three (64.7%) patients were mothers of at least one child and had completed their family planning. Only two null gravida women still wanted a child. Neither the desire for children nor parity were correlated with quality of life at any visit.

## Discussion

The course of AMH before, during and after chemotherapy in breast cancer patients has been addressed in a small number of studies, all of which did not comprise more than 59 patients. Like other authors we found that AMH and AFC levels fell significantly and rapidly from V1 to V2–4, and did not return to pre-chemotherapy levels until the end of the study [25–28]. This drastic change suggests that the majority of primordial follicles are affected by chemotherapy. A clear association was observed between AMH values before chemotherapy and those registered 2 years later. This has been confirmed by Anderson et al., who found that AMH was a valuable indicator of ovarian function and reserve after chemotherapy [18, 29]. The authors also showed that AMH concentrations before chemotherapy predict long-term ovarian function after cytoreductive treatment [30].

In our study, pre-chemotherapy levels of AFC as well as AMH were negatively correlated with age. In addition to AMH, young age was shown to be one of the most important predictors of restored endocrine function and potential fertility in breast cancer survivors [31, 32].

In the present study, ovarian size was investigated as an additional parameter of ovarian activity and proved to be a marker of ovarian endocrine function, possibly even ovarian reserve. Ovarian volume was reduced after the start of chemotherapy, increased later, and was significantly correlated with low AFC, high LH and FSH, and amenorrhea. Ovarian size has been addressed in a small number of published studies. Anderson reported that ovarian size at 12 months after the start of chemotherapy was significantly smaller than the pretreatment size, and added later that ovarian volume was significantly higher in women with regular menstruation after 5 years of chemotherapy than in amenorrhoic ones [18, 30]. Our data revealed no significant association between AMH and the duration of amenorrhea. This may have been because some patients could not be examined on cycle day 3–5 at V1; thus the correct ovarian size may not have been registered at this visit.

Taking a closer look at the endocrine parameters of ovarian function, we registered similar data as those reported earlier [18, 25]. FSH and LH were high, similar to those in postmenopausal women, and E2 was significantly reduced at all visits compared to pre-treatment levels.

LH and FSH levels at V3 and V4 differed in patients receiving different endocrine therapies. Those who received tamoxifen alone had significantly higher levels of gonadotropins than those who were given additional GnRH analogs, thus indicating a potential protective effect of GnRH analogs on fertility.

This question has been controversially discussed in the past. The recent ZORO study comprising 60 women showed no significant difference in amenorrhea between patients treated with or without GnRH agonists [33], whereas the larger POEMS study (257 patients) revealed significantly fewer amenorrhoic patients and more pregnancies among breast cancer survivors in the GnRH agonist group [34]. In a recent meta-analysis as well, more patients who received GnRH agonists had regular menstruation, but the authors were unable to draw final conclusions concerning fertility [35]. The current guidelines of the American Society for Reproductive Medicine recommend the use of GnRH agonists for the preservation of fertility [36].

We observed no association between the type or cumulative dose of chemotherapy and any of the analyzed markers, such as AFC, AMH, ovarian size, the duration of amenorrhea, LH, FSH, or E2. The study with the longest follow-up period so far confirmed the absence of this association at the end of the observation period of 5 years [17, 32].

Notably, 35.4% of our patients had resumed menstruation at month 24 after chemotherapy while 64.6% remained amenorrhoic. The duration of amenorrhea was negatively correlated with the markers of ovarian reserve (AFC and AMH) at nearly all visits, suggesting that regained menstruation is indeed a critical marker of ovarian reserve. Nevertheless, AMH does not predict the recurrence of menstrual bleeding in all cases [25]. We registered no correlation between the duration of amenorrhea and age. A marginal influence of age on amenorrhea has been reported by some groups [30] and contradicted by others [20].

We found striking evidence of the detrimental effect of smoking on ovarian reserve: smokers not only had lower E2 levels, smaller ovaries and a lower AFC than non-smokers 2 years after chemotherapy, but also remained amenorrhoic in large numbers (92%). Smoking not only targets and impairs all stages of reproductive function, but also heightens ovarian aging, possibly resulting in premature menopause [37]. Active smoking is known to be associated with reduced AMH levels in perimenopausal women [38]. This suggests a potential damaging effect of smoking on AFC, thus confirming our data.

Based on the present data we suggest that all young breast cancer patients should receive a basic diagnostic work-up with transvaginal ultrasound and serum analysis before the initiation of chemotherapy. This would serve as a basis for fertility counseling, the options, risks and likelihood of successful conception. Currently a mere 50% of all patients worldwide receive such guidance. As a result, many women start chemotherapy without being aware of the potential loss of fertility [39, 40]. This is alarming in view of the fact that approximately one third of all premenopausal breast cancer patients still wish to have children at the time of diagnosis [40].

Especially young breast cancer patients are at high risk of recurrence and therefore receive aggressive treatment with severe side effects. As revealed by questionnaires, the patients reported various side effects of cytotoxic treatment, such as toxic, psychosocial, and functional ones. We registered no correlation between quality of life and markers of ovarian activity, ovarian reserve or the desire to have children. Nevertheless, unwanted childlessness is defined as a disease by the WHO and must therefore be viewed as a side effect of chemotherapy. Professional counseling could help to diminish this severe side effect and enhance the patients’ quality of life.

## Conclusions

We confirmed the importance of AMH, AFC and age as predictors of ovarian function and reserve in breast cancer patients undergoing chemotherapy. Additionally, smoking seems to be a significant marker in terms of predicting decreased ovarian activity and reserve. More studies will be need to clarify the influence of smoking and other factors on fertility. Reliable information obtained from future studies will help clinicians and women to evaluate treatment options and fertility preservation strategies before the initiation of chemotherapy.

## Additional file

Additional file 1: Questionnaire, designed for this study, containing questions on age, marital status, parity and children, education, pre-existing medical conditions, medication, smoking and alcohol consumption, the ongoing desire to have children, history of miscarriage and abortions, contraceptive methods used, the duration of the menstrual cycle, age at menarche, and when amenorrhea occurred during or after chemotherapy. (DOCX 16 kb)

## Abbreviations

A: Anthracycline-based
AF: Anthracycline-free
AFC: Antral follicle count
AMH: Anti-Müllerian hormone
BMI: Body mass index
CIA: Chemotherapy-induced amenorrhea
CMF: Cyclophosphamide, methotrexate, 5-fluorouracil
Doc: Docetaxel
E2: Estrogen
EC: Epirubicin, cyclophosphamide
FEC: 5-Fluorouracil, epirubicin, cyclophosphamide
FSH: Follicle-stimulating hormone
GnRH: Gonadotropin-releasing hormone
HR: Hormone receptor
IVF: in vitro fertilization
IVM: in vitro maturation
LH: Luteinizing hormone
Pac: Paclitaxel
QL: Quality of life
SPSS: Statistical Package for the Social Sciences
T: Taxanes
TAC: Docetaxel, doxorubicin, cyclophosphamide
V1-4: Visit 1-4
WHO: World Health Organization

## References

[1] JW PE, Fink D, Köchli O (2011). Praxisbuch Gynäkologische Onkologie.

[2] Clarke MJ (2008). WITHDRAWN: multi-agent chemotherapy for early breast cancer. Cochrane Database Syst Rev.

[3] Davies C, Godwin J, Gray R, Clarke M, Cutter D, Darby S, McGale P, Pan HC, Taylor C, Wang YC (2011). Relevance of breast cancer hormone receptors and other factors to the efficacy of adjuvant tamoxifen: patient-level meta-analysis of randomised trials. Lancet.

[4] Early Breast Cancer Trialists' Collaborative Group (EBCTCG). Effects of chemotherapy and hormonal therapy for early breast cancer on recurrence and 15-year survival: an overview of the randomised trials. Lancet. 2005;365(9472):1687–717. 10.1016/S0140-6736(05)66544-0 15894097

[5] Castiglione-Gertsch M, O'Neill A, Price KN, Goldhirsch A, Coates AS, Colleoni M, Nasi ML, Bonetti M, Gelber RD (2003). Adjuvant chemotherapy followed by goserelin versus either modality alone for premenopausal lymph node-negative breast cancer: a randomized trial. J Natl Cancer Inst.

[6] Bernhard J, Luo W, Ribi K, Colleoni M, Burstein HJ, Tondini C, Pinotti G, Spazzapan S, Ruhstaller T, Puglisi F (2015). Patient-reported outcomes with adjuvant exemestane versus tamoxifen in premenopausal women with early breast cancer undergoing ovarian suppression (TEXT and SOFT): a combined analysis of two phase 3 randomised trials. Lancet Oncol.

[7] Weeg N, Shalom-Paz E, Wiser A (2012). Age and infertility: the clinical point of view. Minerva Ginecol.

[8] Cox L, Liu JH (2014). Primary ovarian insufficiency: an update. Int J Womens Health.

[9] Kiechle M (2007). Gynäkologie und Geburtshilfe.

[10] Streuli I, Fraisse T, Pillet C, Ibecheole V, Bischof P, de Ziegler D (2008). Serum antimullerian hormone levels remain stable throughout the menstrual cycle and after oral or vaginal administration of synthetic sex steroids. Fertil Steril.

[11] Jayaprakasan K, Campbell B, Hopkisson J, Johnson I, Raine-Fenning N (2010). A prospective, comparative analysis of anti-Mullerian hormone, inhibin-B, and three-dimensional ultrasound determinants of ovarian reserve in the prediction of poor response to controlled ovarian stimulation. Fertil Steril.

[12] Dittrich R, Maltaris T, Hoffmann I, Oppelt PG, Beckmann MW, Mueller A (2010). Fertility preservation in cancer patients. Minerva Ginecol.

[13] van Disseldorp J, Lambalk CB, Kwee J, Looman CW, Eijkemans MJ, Fauser BC, Broekmans FJ (2010). Comparison of inter- and intra-cycle variability of anti-Mullerian hormone and antral follicle counts. Hum Reprod.

[14] Hansen KR, Hodnett GM, Knowlton N, Craig LB (2011). Correlation of ovarian reserve tests with histologically determined primordial follicle number. Fertil Steril.

[15] Seifer DB, Baker VL, Leader B (2011). Age-specific serum anti-Mullerian hormone values for 17,120 women presenting to fertility centers within the United States. Fertil Steril.

[16] Leader B, Baker VL (2014). Maximizing the clinical utility of antimullerian hormone testing in women's health. Curr Opin Obstet Gynecol.

[17] Kelsey TW, Dodwell SK, Wilkinson AG, Greve T, Andersen CY, Anderson RA, Wallace WH (2013). Ovarian volume throughout life: a validated normative model. PLoS One.

[18] Anderson RA, Themmen AP, Al-Qahtani A, Groome NP, Cameron DA (2006). The effects of chemotherapy and long-term gonadotrophin suppression on the ovarian reserve in premenopausal women with breast cancer. Hum Reprod.

[19] Wallace WH, Anderson RA, Irvine DS (2005). Fertility preservation for young patients with cancer: who is at risk and what can be offered?. Lancet Oncol.

[20] Lee SJ, Schover LR, Partridge AH, Patrizio P, Wallace WH, Hagerty K, Beck LN, Brennan LV, Oktay K (2006). American Society of Clinical Oncology recommendations on fertility preservation in cancer patients. J Clin Oncol.

[21] Das M, Shehata F, Son WY, Tulandi T, Holzer H (2012). Ovarian reserve and response to IVF and in vitro maturation treatment following chemotherapy. Hum Reprod.

[22] Donnez J, Dolmans MM, Pellicer A, Diaz-Garcia C, Sanchez Serrano M, Schmidt KT, Ernst E, Luyckx V, Andersen CY (2013). Restoration of ovarian activity and pregnancy after transplantation of cryopreserved ovarian tissue: a review of 60 cases of reimplantation. Fertil Steril.

[23] Coordinator QL (2001). Quality of live unit, EORTC data center: EORTC manual.

[24] Aaronson NK, Ahmedzai S, Bergman B, Bullinger M, Cull A, Duez NJ, Filiberti A, Flechtner H, Fleishman SB, de Haes JC (1993). The European Organization for Research and Treatment of Cancer QLQ-C30: a quality-of-life instrument for use in international clinical trials in oncology. J Natl Cancer Inst.

[25] Yu B, Douglas N, Ferin MJ, Nakhuda GS, Crew K, Lobo RA, Hershman DL (2010). Changes in markers of ovarian reserve and endocrine function in young women with breast cancer undergoing adjuvant chemotherapy. Cancer.

[26] Henry NL, Xia R, Banerjee M, Gersch C, McConnell D, Giacherio D, Schott AF, Pearlman M, Stearns V, Partridge AH (2013). Predictors of recovery of ovarian function during aromatase inhibitor therapy. Ann Oncol.

[27] Lutchman Singh K, Muttukrishna S, Stein RC, McGarrigle HH, Patel A, Parikh B, Groome NP, Davies MC, Chatterjee R (2007). Predictors of ovarian reserve in young women with breast cancer. Br J Cancer.

[28] Anders C, Marcom PK, Peterson B, Gu L, Unruhe S, Welch R, Lyons P, Behera M, Copland S, Kimmick G (2008). A pilot study of predictive markers of chemotherapy-related amenorrhea among premenopausal women with early stage breast cancer. Cancer Investig.

[29] Anderson RA, Rosendahl M, Kelsey TW, Cameron DA (2013). Pretreatment anti-Mullerian hormone predicts for loss of ovarian function after chemotherapy for early breast cancer. Eur J Cancer.

[30] Anderson RA, Cameron DA (2011). Pretreatment serum anti-mullerian hormone predicts long-term ovarian function and bone mass after chemotherapy for early breast cancer. J Clin Endocrinol Metab.

[31] Faddy MJ, Gosden RG, Gougeon A, Richardson SJ, Nelson JF (1992). Accelerated disappearance of ovarian follicles in mid-life: implications for forecasting menopause. Hum Reprod.

[32] Wallace WH, Kelsey TW (2010). Human ovarian reserve from conception to the menopause. PLoS One.

[33] Gerber B, von Minckwitz G, Stehle H, Reimer T, Felberbaum R, Maass N, Fischer D, Sommer HL, Conrad B, Ortmann O (2011). Effect of luteinizing hormone-releasing hormone agonist on ovarian function after modern adjuvant breast cancer chemotherapy: the GBG 37 ZORO study. J Clin Oncol.

[34] Moore HC, Unger JM, Phillips KA, Boyle F, Hitre E, Porter D, Francis PA, Goldstein LJ, Gomez HL, Vallejos CS (2015). Goserelin for ovarian protection during breast-cancer adjuvant chemotherapy. N Engl J Med.

[35] Munhoz RR, Pereira AA, Sasse AD, Hoff PM, Traina TA, Hudis CA, Marques RJ. Gonadotropin-releasing hormone agonists for ovarian function preservation in premenopausal women undergoing chemotherapy for early-stage breast cancer: a systematic review and meta-analysis. JAMA Oncol. 2015;2(1):1–9. 10.1001/jamaoncol.2015.3251 PMC5003018 26426573

[36] Loren AW, Mangu PB, Beck LN, Brennan L, Magdalinski AJ, Partridge AH, Quinn G, Wallace WH, Oktay K (2013). Fertility preservation for patients with cancer: American Society of Clinical Oncology clinical practice guideline update. J Clin Oncol.

[37] Dechanet C, Anahory T, Mathieu Daude JC, Quantin X, Reyftmann L, Hamamah S, Hedon B, Dechaud H (2011). Effects of cigarette smoking on reproduction. Hum Reprod Update.

[38] Plante BJ, Cooper GS, Baird DD, Steiner AZ (2010). The impact of smoking on antimullerian hormone levels in women aged 38 to 50 years. Menopause.

[39] Peccatori FA, Pup LD, Salvagno F, Guido M, Sarno MA, Revelli A, Piane LD, Dolfin E, Franchi D, Molinari E (2012). Fertility preservation methods in breast cancer. Breast Care (Basel).

[40] Ruddy KJ, Gelber SI, Tamimi RM, Ginsburg ES, Schapira L, Come SE, Borges VF, Meyer ME, Partridge AH (2014). Prospective study of fertility concerns and preservation strategies in young women with breast cancer. J Clin Oncol.

[41] J LG B (2008). Kurzgefasste Statistik für die klinische Forschung, Leitfaden für die verteilungsfreie Analyse kleiner Stichproben.

