# Supplementary material for: Reduced ovarian reserve in young early breast cancer patients: preliminary data from a prospective cohort trial
Source: BMC Cancer. 2017 Sep 6;17:632. doi: 10.1186/s12885-017-3593-x (PMC5588613; doi:10.1186/s12885-017-3593-x)
Supplement: Additional file 1: — Questionnaire, designed for this study, containing questions on age, marital status, parity and children, education, pre-existing medical conditions, medication, smoking and alcohol consumption, the ongoing desire to have children, history of miscarriage and abortions, contraceptive methods used, the duration of the menstrual cycle, age at menarche, and when amenorrhea occurred during or after chemotherapy. (DOCX 16 kb) [file 12885_2017_3593_MOESM1_ESM.docx]

Dear Madam!

You are participating in a study to examine the ovarian function of premenopausal women with breast cancer, who receive chemotherapy.

In this context we would like to ask you to answer some questions:

1. How old are you?
2. What kind of school-leaving qualification do you have?
3. What do you do for a living?
4. What is your marital status?
5. Are you taking any medications on a regular basis? Which ones?
6. Do you have any other diseases?
7. Have you ever had any surgery, especially in the context of wishing to become pregnant?
8. Have you ever given birth to a child?
9. Have you ever had a miscarriage or an abortion?
10. Do you want to become pregnant (now or later)?
11. Have you ever had an unfulfilled wish for a child and was that a reason to seek fertility counseling or even treatment?
12. How have you contracepted until now and for how long?
13. At what age did you have your first menstruation?
14. Please describe your menstrual cycle. How many days are between each bleeding and how many days do you bleed each time?
15. When was the first day of your last menstruation?

Thank your very much for your participation!
